# Low energy Si^+^, SiCH_5_^+^, or C^+^ beam injections to silicon substrates during chemical vapor deposition with dimethylsilane

**DOI:** 10.1016/j.heliyon.2023.e19002

**Published:** 2023-08-06

**Authors:** Satoru Yoshimura, Satoshi Sugimoto, Takae Takeuchi, Kensuke Murai, Masato Kiuchi

**Affiliations:** a Division of Materials and Manufacturing Science, Graduate School of Engineering, Osaka University, 2-1 Yamadaoka, Suita, Osaka, 565-0871, Japan; b National Institute of Advanced Industrial Science and Technology (AIST), 1-8-31 Midorigaoka, Ikeda, Osaka, 563-8577, Japan; c Cerast Laboratory, Tokyo, 154-0011, Japan

**Keywords:** Low energy ion beam, Silicon carbide, Chemical vapor deposition, Dimethylsilane

## Abstract

We found that the atomic-concentration-ratio of carbon to silicon (C/Si ratio) in silicon carbide (SiC) films formed by thermal chemical vapor deposition (CVD) was much greater than 1 when the source gas for CVD was dimethylsilane (DMS). Thus, we tried to change carbon-inclusion levels in the film by injecting some ion beams into a depositing SiC film during the CVD process with DMS. Three ion beams, i.e., Si^+^, SiCH_5_^+^, or C^+^ ions were injected to depositing SiC films. The energy of Si^+^, SiCH_5_^+^, and C^+^ ions was 110 eV. The temperature of the substrate was 800 °C. X-ray diffraction of the deposited films showed that 3C–SiC was included in all three samples. X-ray photoelectron spectroscopy (XPS) showed that the C/Si ratio of the obtained SiC film increased significantly following the Si^+^ or C^+^ ion beam irradiations. The XPS measurements also showed that the C/Si ratio of the SiC film obtained by injecting SiCH_5_^+^ beam during thermal CVD with DMS was lower than that of the SiC film formed by thermal CVD with DMS alone.

## Highlights

• C/Si ratio of SiC films formed by chemical vapor deposition (CVD) with dimethylsilane was much greater than 1.
• We investigated changes of carbon-inclusion levels by the injection of some ion beams to depositing SiC films.
• Three ion beams, i.e., Si^+^, SiCH_5_^+^, or C^+^ were irradiated to a depositing SiC film during the process of CVD.
• We found that excessive carbon-inclusion could be reduced by injecting SiCH_5_^+^ ions.

## 1 Introduction

Silicon carbide (SiC) is attracting attention because of the wide-ranging applicability, especially in the electronics field [1]. SiC is composed of Si and C atoms. Therefore, source materials which include both Si and C atoms are required for the deposition of SiC films.

It was reported that crystalline SiC films were deposited by thermal chemical vapor deposition (CVD) using silane and propane [2]. Meanwhile, methylsilane, trimethylsilane, and hexamehyldisilane have both Si and C atoms. Therefore, methylsilane [[3], [4], [5], [6], [7], [8], [9], [10], [11], [12], [13], [14], [15]], trimethylsilane [16], and hexamethyldisilane [[17], [18], [19], [20], [21], [22], [23], [24], [25], [26], [27], [28], [29]] have been employed in the SiC film formation experiments with various deposition methods such as thermal CVD and plasma-enhanced CVD.

Dimethylsilane (DMS) molecules also have both Si and C atoms. It was reported that SiC films could be produced using DMS [[30], [31], [32], [33], [34], [35], [36]]. When DMS was used for SiC film formations by thermal CVD, the atomic-concentration-ratio of carbon to silicon (C/Si ratio) was much greater than 1 (i.e., excessive carbon-inclusion in SiC) [31,35,36]. The C/Si composition ratio of methylsilane (H_3_SiCH_3_) is 1 and agrees with that of stoichiometric SiC films. By contrast, the C/Si composition ratio of DMS (H_2_Si(CH_3_)_2_) is 2, it seems probable that in the previous experiments [31,35,36] excessive carbon atoms were actually supplied to the substrate. Senthil et al. investigated the absorption kinetics of DMS at a Si substrate and they found enhanced C incorporation as compared with methylsilane by a factor of 1.5 [36].

Properties of non-stoichiometric SiC were quite different from those of stoichiometric SiC and non-stoichiometric SiC films have various potential applications [37]. In this paper, we tried to determine whether carbon-inclusion levels in the film can be changed by injecting some ion beams into a depositing SiC film. Three ion beams, i.e., Si^+^, SiCH_5_^+^, or C^+^ were thus injected to depositing films during thermal CVD with DMS.

## 2 Experimental setup

Experiments were performed using a low energy ion beam device [38]. A source gas was introduced into an ion source, and produced ions in the ion source were extracted by applying 15 kV voltage. Next, the extracted ions were mass-separated. Finally, mass-separated ions were led to a film forming chamber. The ions were decelerated to about 100 eV and injected into a substrate. In this study, Si^+^, SiCH_5_^+^, or C^+^ ions were used as injecting ions.

**Fig. 1:**
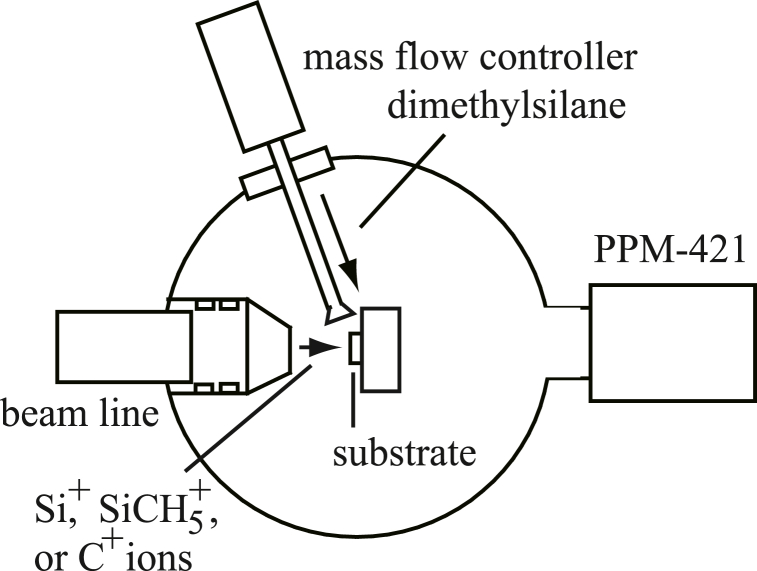
Schematic view of the film forming chamber of the ion beam device.

Fig. 1 is a diagram of the film forming chamber. The degree of base vacuum pressure was 1 × 10^−6^ Pa. A Si (111) substrate was located on a sample holder in the film forming chamber. Natural oxide layer of the Si (111) substrate was removed using diluted hydrogen fluoride before setting on the sample holder. In a previous experiment [35], we tried to form SiC films by CVD with DMS. When the substrate temperature was 800 °C, a crystalline SiC film was found to be formed on a Si substrate. On the contrary, no crystalline SiC film formation was observed in the case of 600 °C. In this paper, the temperature was set to be 800 °C in all trials. The temperature was monitored by an infrared thermometer (CHINO, IR-CAT2CS) during each trial. During ion beam injections, DMS was sprayed onto the substrate. The DMS flow rate (0.3 sccm) was controlled by a mass-flow-controller (HORIBA, SEC-400). The gas pressure in the chamber was 3 × 10^−3^ Pa during film formation trials.

After film formation trials, we analyzed the deposited films with X-ray diffraction (XRD), Fourier transform infrared (FTIR) spectroscopy, X-ray photoelectron spectroscopy (XPS), and Raman spectroscopy.

## 3 Experimental results and discussion

In this paper, Si^+^ and SiCH_5_^+^ ion beams were obtained using methylsilane. Identification of ions produced from methylsilane have been already reported in our previous paper [39]. On the other hand, C^+^ ion beams were produced using CO_2_. The currents of Si^+^ and SiCH_5_^+^ beams were 0.5 and 0.15 μA, respectively. The C^+^ ion beam current was 1.5 μA.

**Fig. 2:**
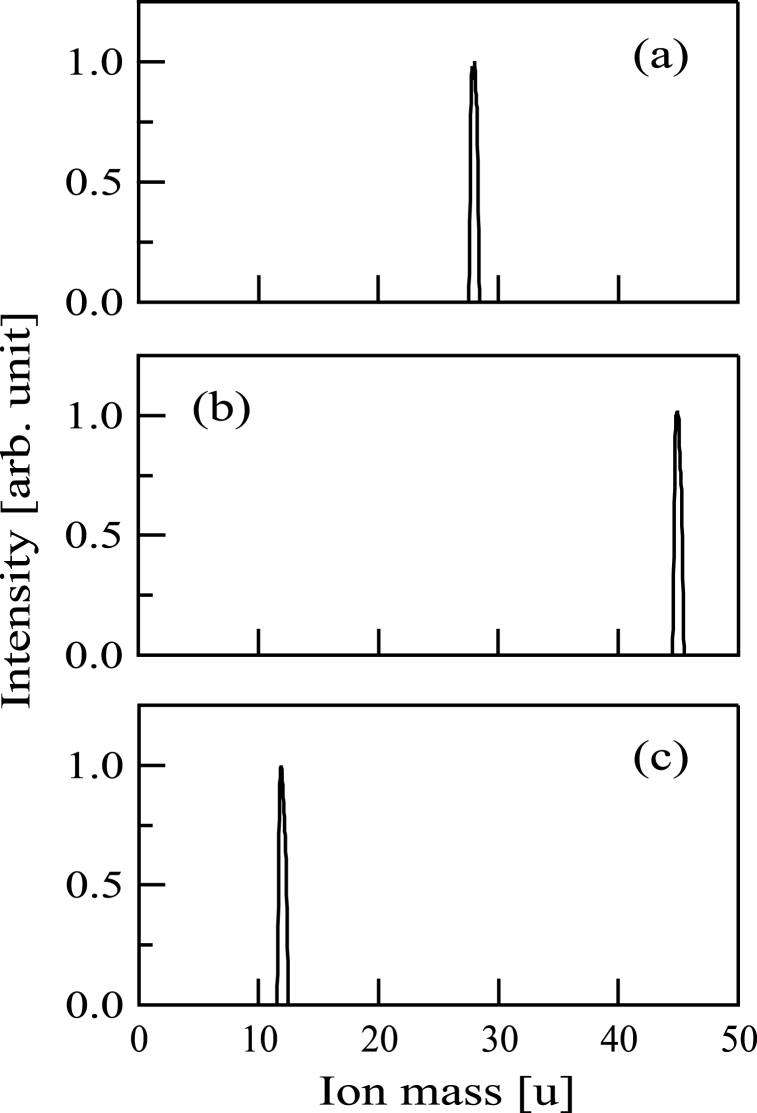
Mass distributions of (a) Si^+^, (b) SiCH_5_^+^, and (c) C^+^ ion beams.

Mass spectra of Si^+^, SiCH_5_^+^, and C^+^ ion beams were obtained by an ion analyzer (Balzers, PPM-421). Fig. 2(a) shows that this is the Si^+^ ion beam because the peak is located at 28 u. The peak in Fig. 2(b) is at 45 u, showing that the beam was composed of SiCH_5_^+^ ions. The peak in Fig. 2(c) is at 12 u (C^+^ ions).

**Fig. 3:**
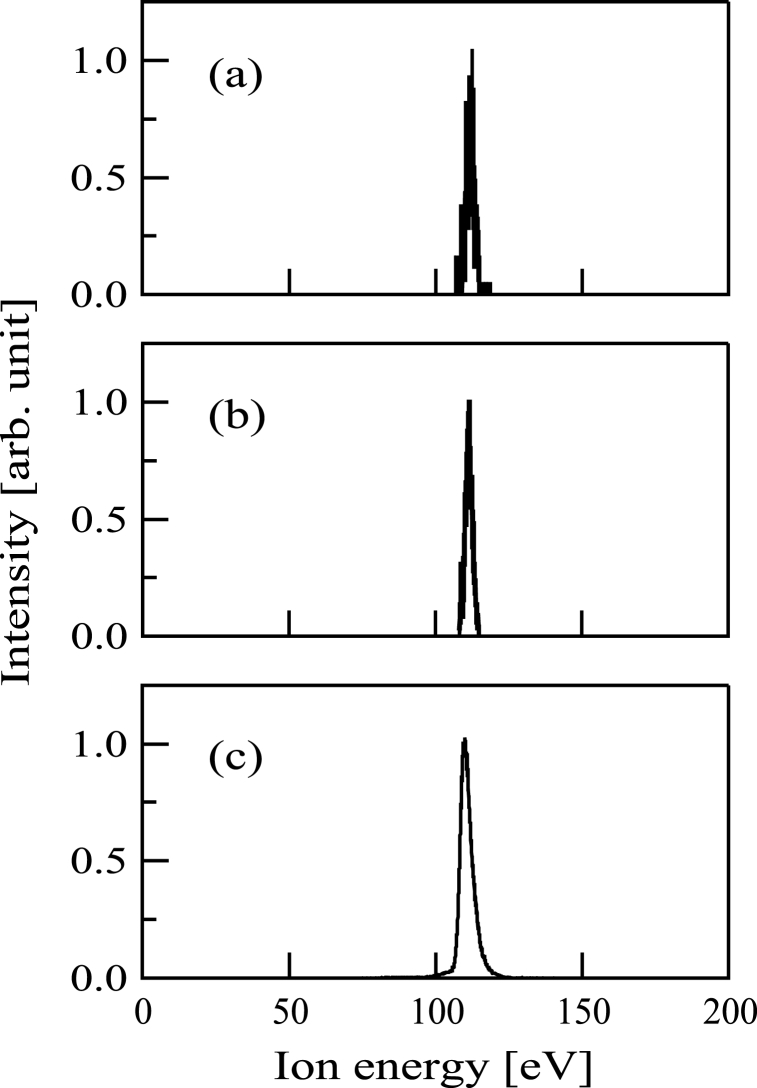
Energy distributions of (a) Si^+^, (b) SiCH_5_^+^, and (c) C^+^ ion beams.

Subsequently, energy spectra of the ion beams were obtained by the PPM-421 analyzer. The energies of the Si^+^, SiCH_5_^+^, and C^+^ beams are shown in Fig. 3(a) and (b), and 3(c). The ion energy was 110 eV in Fig. 3(a)-3(c).

**Fig. 4:**
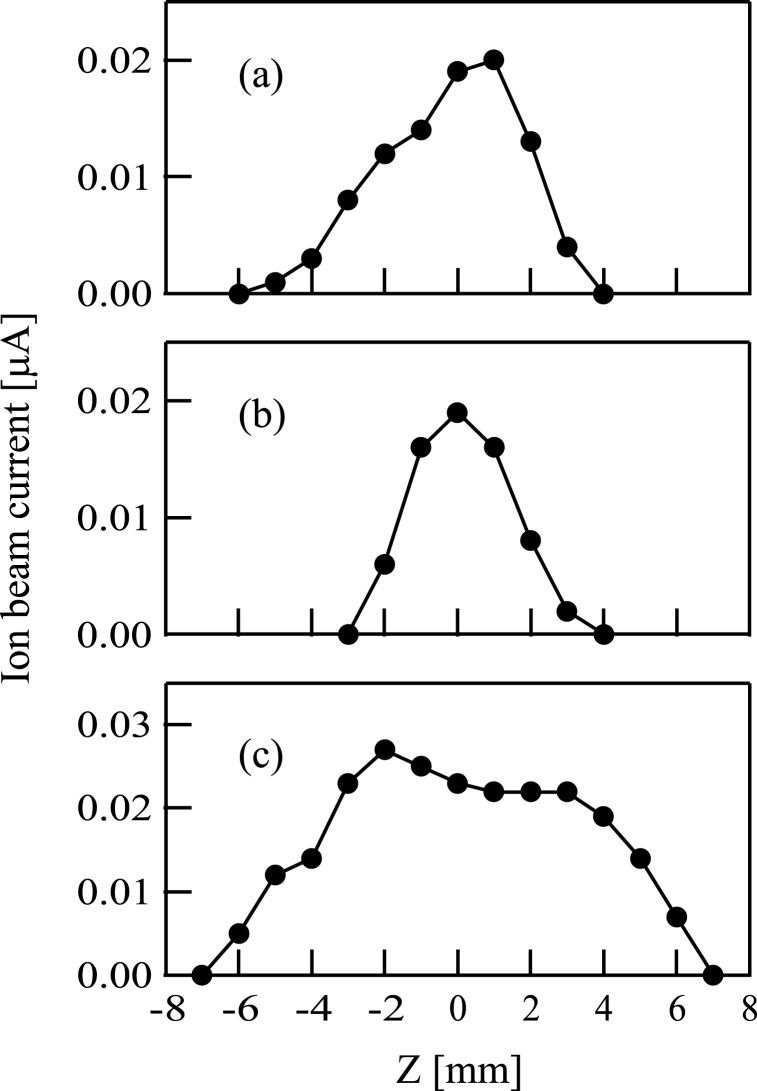
Profiles of (a) Si^+^, (b) SiCH_5_^+^, and (c) C^+^ ion beams.

Profiles of beam intensities were obtained by a Faraday cup with an orifice plate. The beam profiles of Si^+^, SiCH_5_^+^, and C^+^ ion beams are shown in Fig. 4(a) and 4(b), and 4(c). The horizontal axis of Fig. 4(a)-4(c) corresponds to the distance in the vertical direction.

Before the ion beam experiments, DMS was sprayed to a Si substrate without ion beam injections. The duration was 9 h. A film was found to be deposited on the substrate. The thickness was 0.1 μm. The deposition rate was about 0.01 μm/h. The obtained film was analyzed with XRD and XPS. A peak due to 3C–SiC (111) was observed in the XRD pattern obtained with a diffractometer (RIGAKU, RINT2200). XPS spectra were obtained using an XPS device (Kratos, AXIS-165×). The C/Si ratio estimated from the XPS data was 1.88, suggesting that carbon atoms were included excessively, when compared with stoichiometric SiC films.

Then, three trials (a), (b), and (c) were performed. In each trial, a substrate was irradiated with ion beams together with simultaneous spraying of DMS gas. For these trials, (a) Si^+^, (b) SiCH_5_^+^, and (c) C^+^ ion beams were used, respectively. The trials were performed for (a) 9.4, (b) 9, and (c) 8.7 h. Three samples obtained in the trials (a)-(c) were referred to as the samples (a)-(c). After each trial, a film was found to be formed on the substrate. The film was measured with a stylus profiler (KLA-Tencor, P-15). The thickness was 0.1 μm in all three samples (a)-(c). The deposition rate was about 0.01 μm/h in the trials (a)-(c).

**Fig. 5:**
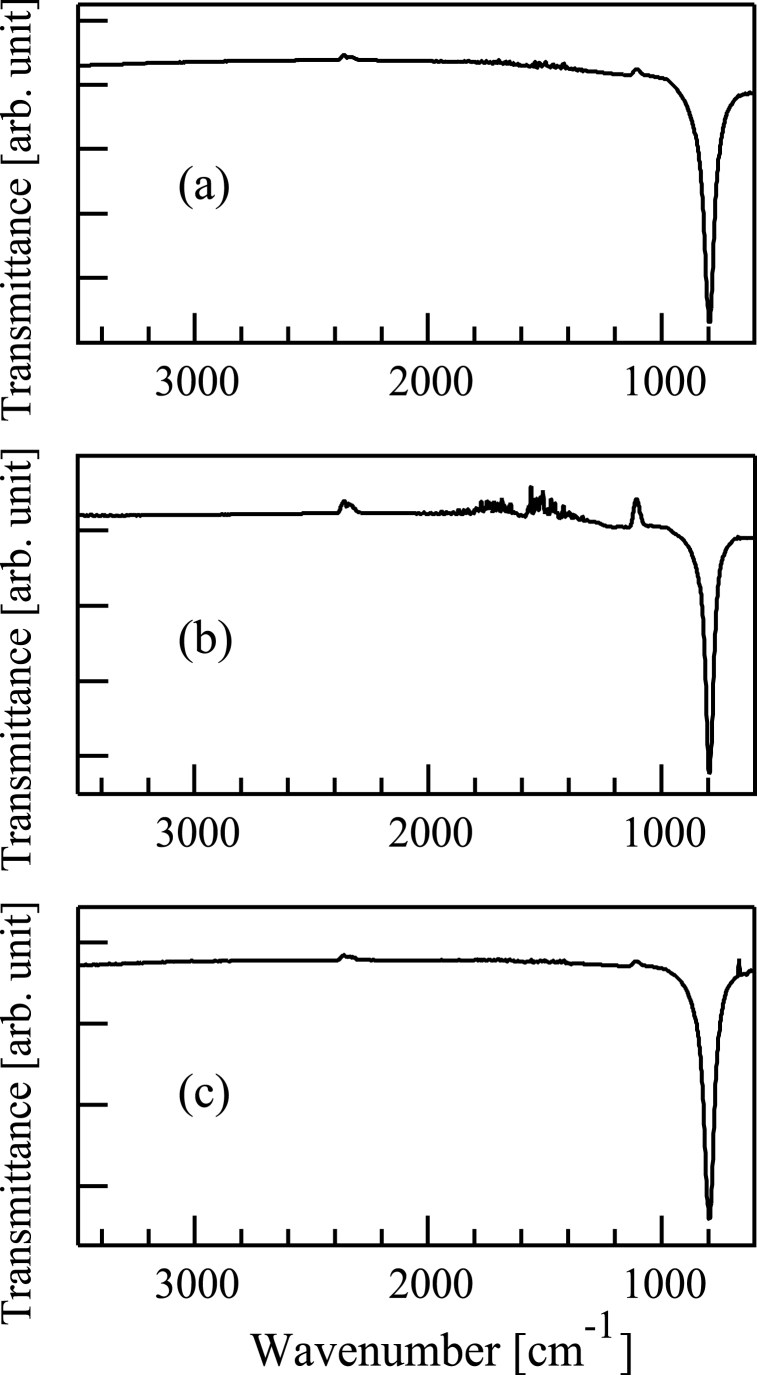
Fourier transform infrared spectrum of a film formed following (a) Si^+^, (b) SiCH_5_^+^, or (c) C^+^ ion beam injection to a substrate in conjunction with dimethylsilane. The substrate was 800 °C in all cases.

Then, the samples were analyzed by FTIR. FTIR spectra were acquired with a spectrometer (Jasco, FT/IR-410). Fig. 5(a)-5(c) show the FTIR spectra of the samples (a)-(c). A peak can be seen at about 800 cm^−1^ in Fig. 5(a)-5(c). This peak corresponds to SiC [40], indicating that SiC films were deposited on the samples (a)-(c).

**Fig. 6:**
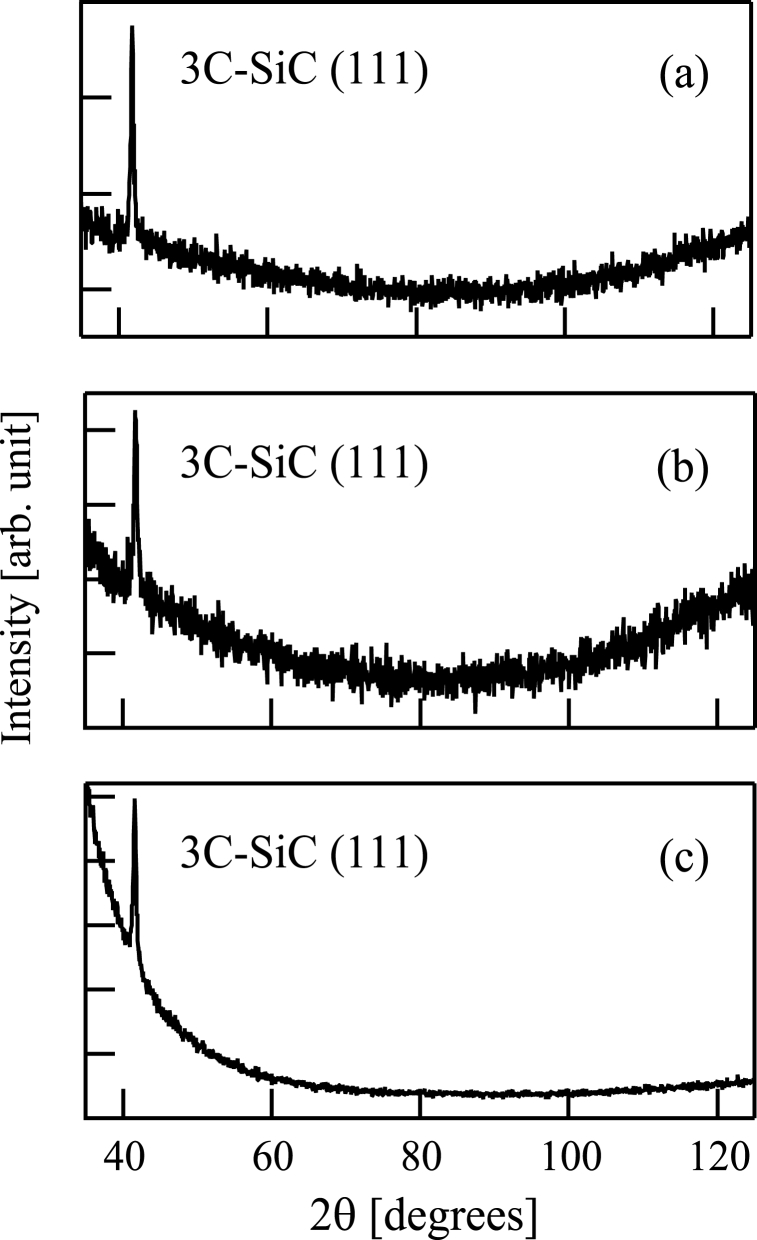
X-ray diffraction pattern (θ-2θ method) of a film formed following (a) Si^+^, (b) SiCH_5_^+^, or (c) C^+^ ion beam injection to a substrate in conjunction with dimethylsilane. The substrate was 800 °C in all cases.

Then, the samples (a)-(c) were assessed by XRD using a diffractometer (RIGAKU, RINT2200) with K_α1_ of cobalt (λ = 1.78892 Å). Fig. 6(a)-6(c) show the XRD results (θ-2θ method). A 3C–SiC (111) peak can be seen in Fig. 6(a)-6(c). Fig. 6(a)-6(c) show that crystalline SiC was included in the deposited films on the samples (a)-(c).

**Fig. 7:**
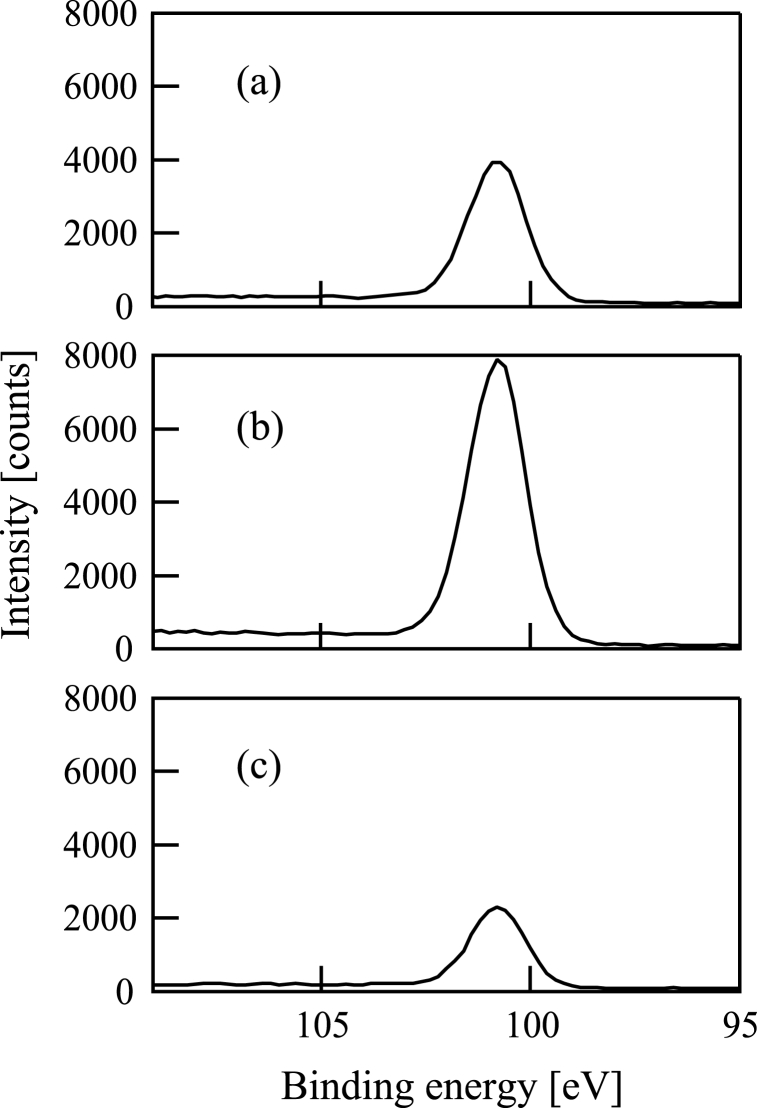
Si2p X-ray photoelectron spectrum of a film formed following (a) Si^+^, (b) SiCH_5_^+^, or (c) C^+^ ion beam injection to a substrate in conjunction with dimethylsilane. The substrate was 800 °C in all cases.

**Fig. 8:**
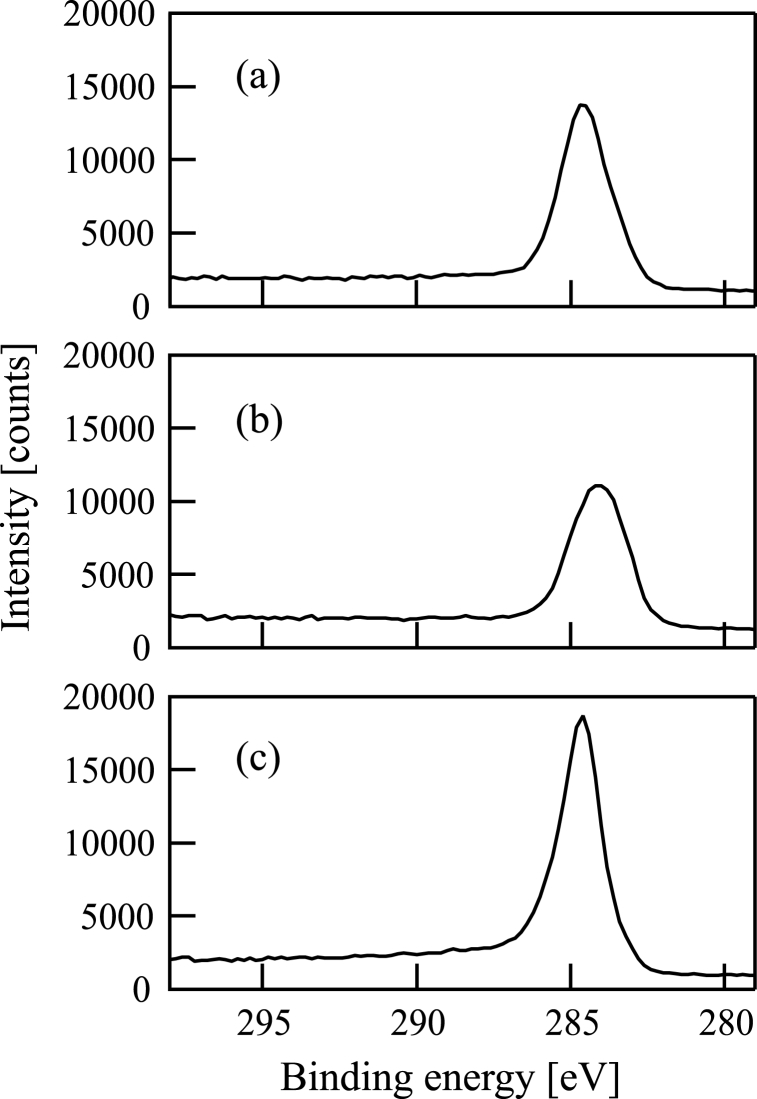
C1s X-ray photoelectron spectrum of a film formed following the injection of (a) Si^+^, (b) SiCH_5_^+^, or (c) C^+^ ion beam injection to a substrate in conjunction with dimethylsilane. The substrate was 800 °C in all cases.

Subsequently, XPS spectra of the samples were obtained using an XPS device (ULVAC-PHI, ESCA-3057). Fig. 7(a)-7(c) and Fig. 8(a)-8(c) show Si2p and C1s XPS spectra of the samples (a)-(c), respectively. There is an obvious Si2p peak in each figure of Fig. 7(a)-7(c) at the binding energy corresponding to SiC (100.8 eV). The full widths at half maximum (FWHMs) of the Si2p XPS spectra are 1.6 eV in Fig. 7(a)-7(c). The C1s peaks in Fig. 8(a)-8(c) are at (a) 284.6, (b) 284.1, and (c) 284.6 eV. FWHMs of the C1s XPS spectra are (a) 1.8, (b) 2.2, and (c) 1.5 eV in Fig. 8(a)-8(c).

**Fig. 9:**
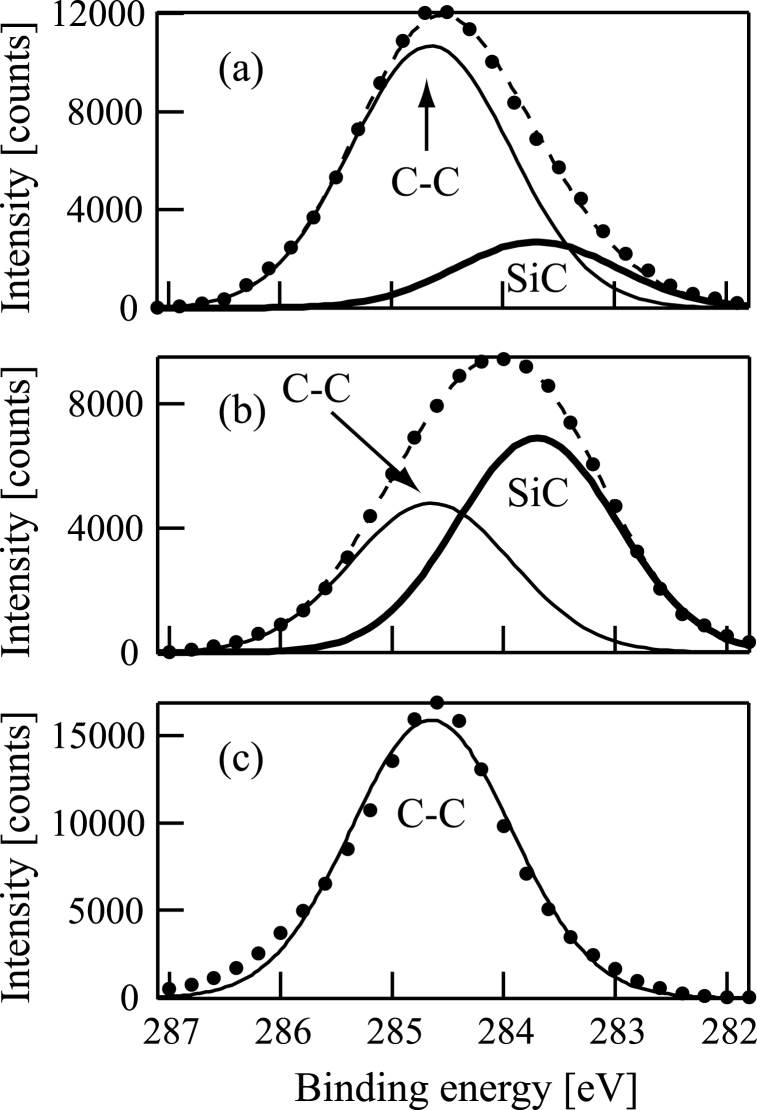
Curve fitting of the C1s peak of a film formed following (a) Si^+^, (b) SiCH_5_^+^, or (c) C^+^ ion beam injection to a substrate in conjunction with dimethylsilane. The substrate was 800 °C in all cases. In figures (a)–(c), the C1s spectra are shown by closed circles. In figures (a) and (b), the thin dashed line corresponds to the fitted curve. The thin solid line corresponds to the C–C bond in figures (a)–(c). The thick solid line corresponds to the SiC bond in figures (a) and (b).

Then, curve fittings of the XPS data were performed. The C1s peaks in Fig. 8(a)-8(c) were decomposed with the least-squares method. The results are shown in Fig. 9(a)-9(c). Fig. 9(a) and (b) show that the samples (a) and (b) contain both C–C bond (284.6 eV) and SiC bond (283.7 eV). Fig. 9(a) shows that the intensity of the C–C bond peak is much larger than that of the SiC bond. On the contrary, the intensity of the C–C bond peak is smaller than that of the SiC bond in Fig. 9(b). By contrast, Fig. 9(c) shows that the C1s peak obtained from the sample (c) can be fitted by the C–C bond curve alone.

Atomic concentrations in the films can be estimated using the XPS data with the accuracy of 1 atomic%. The estimation showed that the C/Si ratios were (a) 3.45, (b) 1.49, and (c) 7.94 for the samples (a)-(c). In all samples (a)-(c), the C/Si ratios were greater than 1, suggesting that carbon atoms were included excessively in the films. In particular, the C/Si ratio in the sample (c) was quite large. By contrast, the C/Si ratio was relatively small in the sample (b).

**Fig. 10:**
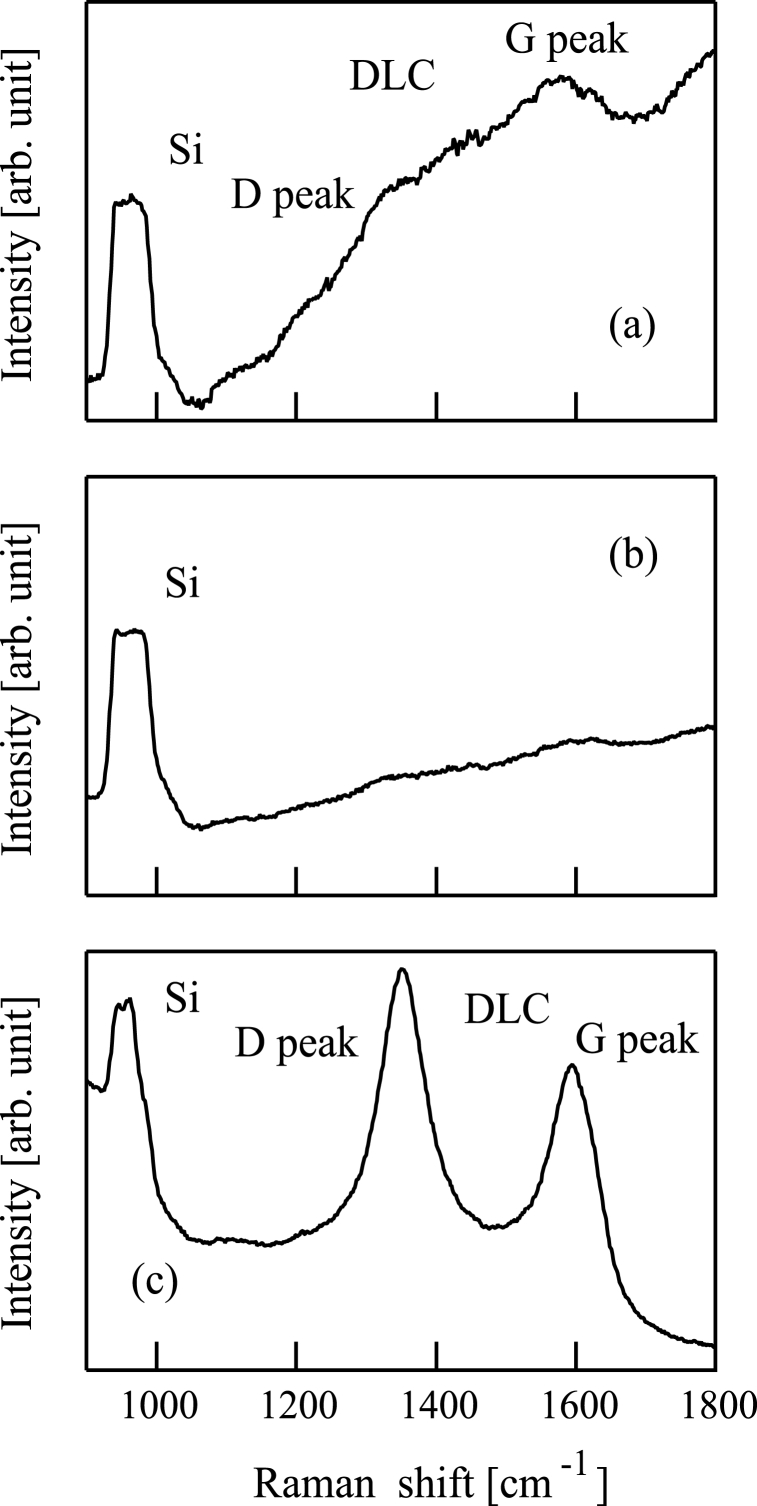
Raman spectroscopy spectrum of a film formed following (a) Si^+^, (b) SiCH_5_^+^, or (c) C^+^ ion beam injection to a substrate in conjunction with dimethylsilane. The substrate was 800 °C in all cases.

Raman spectroscopy was carried out for the samples (a)-(c) with an instrument (HORIBA, LABRAM-HR800WH). Raman spectra are shown in Fig. 10(a)-10(c). In Fig. 10(a)-10(c), a peak at 950 cm^−1^ corresponds to the second-order Raman spectra for Si substrates [41].Fig. 10(a)–(c) show that the samples (a) and (c) contained diamond-like carbon (DLC) because strong graphite (G) and disorder (D) peaks can be seen in Fig. 10(a) and 10(c). By contrast, no strong DLC peaks can be seen in Fig. 10(b).

In the present study, we found that the C/Si ratio of the film formed by thermal CVD with DMS alone was 1.88. Firstly, we tried to supplement additional Si atoms to a depositing SiC film by injecting Si^+^ ions for the reduction of the carbon ratio of the film. However, the C/Si ratio was 3.45 in the sample (a). Contrary to our expectation, the carbon ratio of the obtained film did not decrease, but increased significantly by the Si^+^ ion beam irradiation. This result suggests that a part of Si atoms in the depositing SiC film was ejected by the injection of Si^+^ ions. Based on this result, we speculate as follows: If C^+^ ions instead of Si^+^ ions were irradiated to a depositing SiC film, a part of C atoms in the film would be expelled and the carbon ratio in the film would decrease. Therefore, in another trial, C^+^ ions were irradiated to a depositing film [sample (c)]. However, the experimental results showed that in the sample (c) the C/Si ratio was much greater than that obtained by thermal CVD with DMS alone. This finding clearly suggests that most of injected C^+^ ions could have been incorporated into the depositing film.

Yamamura et al. [42] and Roth et al. [43] reported self-sputtering yields of graphite by C^+^ ions. They showed that self-sputtering yields of graphite by C^+^ ions were less than 0.1 when the injected C^+^ energy was in the range of 70–200 eV [42,43]. When C^+^ energy was 110 eV, most of injected C^+^ ions were deposited on the graphite substrate. Although the SiC sputtering yields by C^+^ ion injections have not been determined, it seems probable that the C^+^ ions injected to a depositing SiC film were incorporated into the film.

The evaluated atomic-concentration-ratio (3.45) of the sample (a) suggests that when 110 eV Si^+^ ions were injected to SiC films to be deposited by CVD with DMS, a part of Si atoms in the depositing SiC film was probably eliminated from the film. Unfortunately, the SiC sputtering yields by Si^+^ ion injections have not been reported. Based on the large C/Si ratio of the sample (a), however, we think that Si atoms in the deposited SiC film were sputtered by the Si^+^ ion injections.

Then, we tried to measure self-sputtering yields of Si. A quartz crystal microbalance (QCM) system (ULVAC, CRTM-9000) was used for the measurement. The temperature of the QCM substrate was set to be room temperature. Prior to self-sputtering yield measurements, a Si film was prepared on the substrate by injecting low energy Si^+^ ion beams to the QCM substrate surface. The QCM controller showed that the deposited film thickness was 0.03 μm. Then, 110 eV Si^+^ ion beams were injected to the film. The change of Si film thickness was measured by the QCM controller. The number of incident Si^+^ ions was measured by a Faraday cup. The self-sputtering yield of Si by 110 eV Si^+^ ion injections was evaluated to be 0.21.

On the other hand, SiCH_5_^+^ ions were injected in the trial (b). In the sample (b), the C/Si ratio was 1.49, which was smaller than that (1.88) obtained by thermal CVD with DMS alone. This result indicates that the SiCH_5_^+^ ion injection is available for decreasing carbon-inclusion levels in the SiC film to be deposited by CVD with DMS.

In our previous study [35], we found that Ar^+^ ion beam injection had an effect similar to the present sample (b). Ar^+^ ion injection during CVD with DMS decreased the C/Si ratio to 1.36 [35]. The mass of Ar^+^ ions (40 u) is similar to that of SiCH_5_^+^ ions (45 u). Thus, this probably explains the reason why SiCH_5_^+^ ion beam injections have a similar reducing effect of carbon-inclusion levels in the deposited SiC film. The ion beam intensity of SiCH_5_^+^ was only 0.15 μA and was much smaller than that (2 μA) of Ar^+^ ion beam in the previous study [35]. If we increased the SiCH_5_^+^ ion beam intensity further, carbon atoms excessively included in the film would decrease. However, it was difficult for us to increase the beam intensity of SiCH_5_^+^ ions further.

Another possible explanation is available for the reason why SiCH_5_^+^ ion beam injections have a reducing effect of carbon-inclusion levels in the deposited SiC film. In the case of SiCH_5_^+^ ion beam, the injecting energy of 110 eV was distributed to the constituent atoms. The injecting energies of Si and C atoms were evaluated to be 68 and 29 eV, respectively. In this energy range (68 and 29 eV), injected Si and C atoms seemed to be deposited without sputtering the film [42]. Therefore, if 110 eV SiCH_5_^+^ ions were injected into the depositing film, injected silicon and carbon atoms would be incorporated in the film and the C/Si ratio of the film could result in a value between the composition ratio of DMS (C/Si = 2) and that of injecting SiCH_5_^+^ ions (C/Si = 1).

## 4 Conclusion

We found that the carbon to silicon ratio of the deposited SiC film was C/Si = 1.88 when DMS was employed in the thermal CVD experiments. In this paper, we tried to determine whether carbon-inclusion levels in the film can be changed by injecting some ions into a depositing SiC film during the thermal CVD process with DMS. For this purpose, Si^+^, SiCH_5_^+^, and C^+^ ions were employed. The energy of Si^+^, SiCH_5_^+^, and C^+^ ions was 110 eV each. The substrate temperature was 800 °C. Films were formed on the substrates which had been additionally disposed to (a) Si^+^, (b) SiCH_5_^+^ and (c) C^+^ ion beams. FTIR and XRD results of the deposited films show that 3C–SiC was included in all three samples (a)-(c). XPS results showed that the C/Si ratio increased significantly following the Si^+^ or C^+^ ion beam irradiation in the samples (a) and (c). By contrast, in the sample (b) the C/Si ratio was relatively small, which was smaller than that obtained by thermal CVD with DMS alone. This result implies that SiCH_5_^+^ ion injections were useful for decreasing carbon-inclusion levels in the SiC films formed following CVD with DMS.

## Author contribution statement

Satoru Yoshimura: Conceived and designed the experiments; Performed the experiments; Wrote the paper. Satoshi Sugimoto; Kensuke Murai: Performed the experiments; Contributed reagents, materials, analysis tools or data. Takae Takeuchi; Masato Kiuchi: Analyzed and interpreted the data; Contributed reagents, materials, analysis tools or data. Data availability statement: Data included in article/supp. Material/referenced in article.

## Funding statement

This research did not receive any specific grant from funding agencies in the public, commercial, or not-for-profit sectors.

## Additional information

No additional information is available for this paper.

## Declaration of competing interest

The authors declare that they have no known competing financial interests or personal relationships that could have appeared to influence the work reported in this paper.
